# Influence of hydrolysis rate of urea on ruminal bacterial diversity level and cellulolytic bacteria abundance in vitro

**DOI:** 10.7717/peerj.5475

**Published:** 2018-08-17

**Authors:** Pengpeng Wang, Shengguo Zhao, Xuemei Nan, Di Jin, Jiaqi Wang

**Affiliations:** Chinese Academy of Agricultural Sciences, State Key Laboratory of Animal Nutrition, Institute of Animal Science, Beijing, People’s Republic of China

**Keywords:** Urea, Ammonia, Acetohydroxamic acid, Cellulolytic bacteria, Bacterial community

## Abstract

The objective of this experiment was to evaluate the effects of urea hydrolysis rate on ruminal bacterial diversity level and cellulolytic bacteria abundance in vitro. To control urea hydrolysis rate, urea and urease inhibitor (acetohydroxamic acid, AHA) were supplemented to a 2 × 2 factorial design, with urea supplemented at 0 or 20 g/kg dry matter (DM) of substrate, and AHA equivalent to 0 or 450 mg/kg DM of substrate. Ruminal fluid was collected from three Chinese Holstein dairy cows, fed a TMR, and incubated at 39 °C for 12 h after the addition of urea and AHA. Rumen fermentation parameters, which indicated the rate of ammonia formation (including ammonia-nitrogen (NH_3_-N) and urea-nitrogen concentrations, urease activity, and microbial crude protein) were measured by chemical analysis. Bacterial diversity was analyzed by denaturing gradient gel electrophoresis (DGGE). Total bacteria and cellulolytic bacteria abundance was detected by quantitative PCR. Results showed that AHA addition significantly decreased the rate of ammonia formation when urea was supplemented. Urea and AHA supplementation significantly increased the bacterial community diversity level according to the Shannon–Weiner index of 16S DGGE images. Furthermore, ruminal bacterial profiles were separated by ammonia release rate when urea was supplemented, according to the DGGE and hierarchical cluster analysis. Urea supplementation reduced the abundance of cellulolytic bacteria, such as *Ruminococcus albus*, *R. flavefaciens*, *Fibrobacter succinogenes*, and *Butyrivibrio fibrosolvens*, but inhibition of urea hydrolysis by AHA addition alleviated the reductions during the early period of incubation. In conclusion, slow release of ammonia induced by urease inhibitor influenced the ruminal bacterial diversity level and lessened the inhibition of total bacteria growth at the incubation of 12 h and *F. succinogenes* during the early period of incubation.

## Introduction

During the early and middle lactation periods in dairy cows, high dietary energy, and crude protein (CP) are required to maintain high milk production. Urea, a kind of non-protein nitrogen, is usually supplemented to the diets of dairy cows, in order to compose microbial crude protein (MCP), and reduce the cost of feeding (Kertz, 2010). However, rapid hydrolysis of dietary urea by bacteria in the rumen easily leads to a dramatic increase in the concentration of ammonia in the first few hours after feeding. The detrimental effects of oversupply of ammonia (derived from dietary urea) result in not only decreased energy and protein availability (an asynchronous supply of nitrogen and energy for microbial protein synthesis), but also ammonia toxicity in dairy cows (Recktenwald et al., 2014).

Various strategies are implemented to reduce the rapid hydrolysis of dietary urea into ammonia in the rumen, such as the application of urease inhibitor to diet (Upadhyay, 2012). It is reported that urease inhibitor can effectively delay urea hydrolysis (slow release urea) and decrease the rate of ammonia formation in the rumen without a negative effect on nitrogen metabolism (Zhang, Shan & Bao, 2002). In addition, slow release urea can increase milk fat, protein, and milk energy output of dairy cows during lactation period (Highstreet et al., 2010). Moreover, the risk for ammonia toxicity is reduced when Holstein steers are fed at higher concentrations of urea coupled with urease inhibitor (Holder et al., 2013). However, there is limited information on rumen bacterial population induced by slow hydrolysis of urea.

Ruminal bacteria, especially ruminal cellulolytic bacteria, including *Ruminococcus albus*, *R. flavefaciens*, and *Fibrobacter succinogenes*, synthesize enzymes (such as glutamate dehydrogenase, glutamine synthetase, and glutamate synthetase) that can incorporate ammonia into carbon skeleton to form different amino acids for protein synthesis and bacterial growth (Antonopoulos et al., 2003; Ataşoğlu et al., 1999). It was recently reported that greater amounts of ammonia by feeding urea inhibited some ruminal cellulolytic bacteria in Nellore steers (Granja-Salcedo et al., 2016). Therefore, our hypothesis was that reducing the rate of dietary urea hydrolysis in the rumen may ameliorate the negative effect of high levels of rumen ammonia on rumen bacteria, especially on rumen cellulolytic bacteria. Thus, the objective of the current study was to evaluate the influence of hydrolysis rate of urea on ruminal bacterial diversity level and cellulolytic bacteria abundance in vitro.

## Materials and Methods

All procedures, including the care of animals and collection of ruminal fluid, were approved by the Animal Care and Use Committee for Livestock issued by the Institute of Animal Science, Chinese Academy of Agricultural Sciences (Beijing, China).

### Experimental design

The experimental design was a 2 × 2 factorial arrangement with urea equivalent to 0 and 20 g/kg of substrate (based on dry matter, DM), and with acetohydroxamic acid (AHA) at 0 and 450 mg/kg DM. The AHA was purchased from a commercial company (Xi’an Jingxihua Limited Company, Xi’an, China). The maximal recommended dose was used. To name the treatments, U and UI were represented of urea and urease inhibitor, respectively. Thus, the four treatments were U0UI0, U0UI450, U2UI0, and U2UI450. The experiment was separately run three times on different days of collecting mixed rumen fluids, and each treatment was applied in triplicate at each experimental day.

### In vitro batch fermentation

On each experimental day, ruminal fluid was collected from three ruminally cannulated Chinese Holstein dairy cows approximately 4 h after feeding a total mixed ration. Ruminal fluid from the three cows was combined and rapidly filtered through four layers of sterile cheesecloth into an Erlenmeyer flask containing buffer solution (1:2, v/v) at 39 °C under a continuous flow of CO_2_. The buffer solution was described by Lowe (1985) with some modification (per liter): 0.625 ml of trace elements solution which contained 0.0125 g CaCl_2_ · 2H_2_O, 0.0100 g FeCl_3_ · 6H_2_O, 0.0125 g MnCl_2_ · 4H_2_O, 0.0074 g ZnCl_2_ · H_2_O, 0.0013 g CoCl_2_ · 6H_2_O, and 0.0016 g CuCl_2_ · 2H_2_O, 250 ml of buffer which contained 9.75 g NaHCO_3_, 250 ml of mineral solution which contained 3.59 g Na_2_HPO_4_ · 12H_2_O, 1.55 g KH_2_PO4, 0.15 g MgSO_4_ · 7H_2_O, 1.25 ml of resazurin 0.1% (w/v), and two ml of reducing solution which contained 0.5 g Na_2_S · 9H_2_O. The buffered ruminal fluid (75 ml) with initial pH 6.8 was anaerobically dispensed into a serum bottle (100 ml) containing 1.0 g ground basic diet with the appropriate dosages of urea or AHA or both. The basic diet was formulated the same as the total mixed ration for ruminal fluid donors, and contained (per kg): 177 g alfalfa hay, 175 g corn silage, 51 g oaten hay, 56 g cotton feed, 37 g apple pulp, 67 g sugar beet pulp, 27 g molasses (30%), 181 g steam-flaked corn, 56 g soybean skin, 65 g soybean meal, 39 g extruded soybean, 25 g dried distiller’s grains, 26 g double-low rapeseed meal, 2.75 g Ca(HCO_3_)_2_, 2.75 g CaCO_3_, 3.5 g NaCl, and 6.0 g NaHCO_3_, with the following chemical composition (per kg DM): 165 g CP, 355 g neutral detergent fiber, 217 g acid detergent fiber, and 65 g ether extract. There were 72 bottles (four treatments × three replicates × six sampling time points) used in total. Bottles were purged with oxygen free CO_2_ before being sealed with butyl rubber stoppers plus crimped aluminum seals. Incubation was conducted at 39 °C for 12 h in an incubator with intermittent manual exhaust by inserting syringe needle.

### Sampling and chemical analysis

After incubation of 0.5, 1, 2, 4, 6, and 12 h, fermentation was terminated by placing 12 bottles (four treatments × three replicates) on ice in each sampling time point. Fresh culture fluids were dispensed immediately into different centrifuge tubes and frozen at −20 or −80 °C for further analysis. Ammonia-nitrogen (NH_3_-N) concentration was determined using the sodium salicylic acid and hypochlorous acid spectrophotometric method (Feng & Gao, 1993). Urea-nitrogen (Urea-N) concentration was determined by the diacetyl monoxime method using a commercial kit (Nanjing Jiancheng Co., Nanjing, China) following the manufacturer’s protocol. The measurement of urease activity was described by Yu et al. (2015) with a few modification. In brief, urease activity in 0.5 ml cell-free extracts was determined by adding, in succession, 0.5 ml of urea (50 mmol) and incubated at 37 °C for 30 min, 1.5 ml of phenol-sodium nitroprusside, and 1.5 ml of NaClO–NaOH. The tubes containing the mixed solutions were incubated at 37 °C for 30 min, and then measured by spectrophotometry at 625 nm. One unit of urease activity was defined as that producing 1.0 nmol NH_3_-N per mg of crude enzyme protein in 1 min. After incubation, a eight ml sample of bottle content was collected, and strained through four layers of cheesecloth to separate liquid and solid phases. The solid phase was washed with a prewarmed (39 °C) and oxygen-free saline solution twice, and the eluent was added to the liquid phase. The liquid phase was centrifuged at 500×*g* for 15 min under 4 °C to remove feed particles and protozoa, and the supernatant was centrifuged at 22,000×*g* for 20 min under 4 °C to obtain ruminal bacteria. Ruminal MCP synthesis was estimated by the ratio of purines to isolated bacterial nitrogen (Makkar & Becker, 1999; Zinn & Owens, 1986).

### Microbial DNA extraction

Samples for genomic DNA extraction were collected immediately after incubation of 0.5, 4, and 12 h and preserved at −80 °C. Genomic DNA extraction was carried out using the modified cetyltrimethylammonium bromide method of Brookman & Nicholson (2005), combined with bead beating. The quality of DNA was visualized using a 1% (w/v) agarose gel electrophoresis, and the concentration of DNA was measured at an absorbance of 260 and 280 nm using NanoDrop ND1000 spectrophotometer (NanoDrop Technologies Inc., Wilmington, DE, USA). The DNA samples were stored at −20 °C until analysis.

### PCR and denaturing gradient gel electrophoresis

Primers used to amplify the V3 region of the 16S rRNA gene were GC-338F: (GC clamp-ACTCCTACGGGAGGCAGCAG) and 533R (TTACCGCGGCTGCTGGCAC) (Huse et al., 2008). The PCR reaction solution (50 μl in total) contained one μl of genomic DNA template (diluted to 25 ng/μl), 0.8 μl of Ex Taq Hot Start Version (TaKaRa Bio, Otsu, Japan), five μl of 10 × Ex Taq Buffer (TaKaRa Bio, Otsu, Japan), five μl of dNTP (TaKaRa Bio, Otsu, Japan), two μl of each primer (10 μM), and 34.2 μl of ddH_2_O. The PCR amplification was performed in a Thermo cycler (Biometra, Göttingen, Germany) using the following program: initial denaturation for 3 min at 94 °C; 10 cycles of 94 °C for 30 s, 60 °C for 30 s, decreasing 0.5 °C every cycle, and 72 °C for 30 s; 20 cycles of 94 °C for 30 s, 56 °C for 30 s, and 72 °C for 30 s; and finally, holding at 72 °C for 5 min. The PCR products were confirmed by electrophoresis on a 1.5% (w/v) agarose gel stained with 5% GoldView solution. Before denaturing gradient gel electrophoresis (DGGE) analysis, the PCR products of samples from three separate days for the same treatment were mixed together as one sample, so finally there were 8 PCR products (4 treatment × 2 sampling time points (after incubation of 4 and 12 h in order to verify the alteration of ruminal bacterial diversity level in the stable period)) subjected to DGGE analysis using the DCode Universal Mutation Detection System (Bio-Rad Laboratories, Inc., Hercules, CA, USA). Gradient gels containing 8% (w/v) polyacrylamide (37.5:1 acrylamide/bis-acrylamide) were prepared with 40–60% urea formamide denaturant. The 20–25 μl each mixed PCR products sample with DNA of 200 ng was used for gel loading. The electrophoresis was run at 85 V for 16 h at 60 °C. The DNA bands in the gels were visualized by silver staining according to the procedures described by Creste, Neto & Figueira (2001) and photographed with a Umax Scanner (GS-800; Bio-Rad Laboratories, Inc., Hercules, CA, USA). The density of each PCR–DGGE band was obtained using Quantity One 4.6.2 software (Bio-Rad Laboratories, Inc., Hercules, CA, USA), and the data were subjected to hierarchical cluster analysis using R software with the hclust package (Sørlie et al., 2001). The diversity index of Shannon–Weiner was calculated using the band optical density, which was determined by Quantity One 4.6.2 software (Bio-Rad Laboratories, Inc., Hercules, CA, USA).

### Quantitative real time PCR analysis

The populations of total bacteria and ruminal cellulolytic bacteria were quantified using SYBR Green-based quantitative real time PCR (qPCR) in an ABI 7500 Real Time PCR System (Applied Biosystems, Foster City, CA, USA). The primers are shown in Table 1. The standard curve between quantification cycle (Cq) value and plasmid copy number was constructed based on serial dilutions of the specific plasmid DNA containing the 16S rRNA sequence of those targeted bacteria. The qPCR mixture contained 5.0 μl of SYBR Premix Ex Tag (2×) (TaKaRa Bio, Otsu, Japan), 0.2 μl of ROX Reference Dye II (50×) (TaKaRa Bio, Otsu, Japan), 0.4 μl of each primer (10 μM), one μl of template (10 ng/μl), and three μl of ddH_2_O. The qPCR program consisted of the following: 95 °C for 30 s; 40 cycles of 95 °C for 5 s, 60 °C for 34 s (with fluorescence collection). Both standard and DNA sample were routinely run in triplicates using the same master mix in the same PCR plate. A negative blank (without DNA template) was also run for each primer pair. The copies of total bacteria and ruminal cellulolytic bacteria were determined by relating their Cq values to their standard curves, respectively. Only the data of qPCR efficiencies between 90% and 105% were used for analysis (Zhou, Hernandez Sanabria & Guan, 2009). Data were analyzed using the ABI 7500 SDS software (version 2.3; Applied Biosystems, Foster City, CA, USA). The absolute populations were expressed as copies per milliliter of culture samples.

**Table 1:** The primers used in qPCR.

Item	Primer sequence (5′–3′)	Product size (bp)	Efficiency (%)	Reference
Total bacteria	F: CGGCAACGAGCGCAACCC R: CCATTGTAGCACGTGTGTAGCC	146	92.3	Denman & McSweeney (2006)
*Ruminococcus albus*	F: CCCTAAAAGCAGTCTTAGTTCG R: CCTCCTTGCGGTTAGAACA	176	93.1	Koike & Kobayashi (2001)
*Ruminococcus flavefaciens*	F: GAACGGAGATAATTTGAGTTTACTTAGG R: CGGTCTCTGTATGTTATGAGGTATTACC	132	94.8	Denman & McSweeney (2006)
*Fibrobacter succinogenes*	F: GTTCGGAATTACTGGGCGTAAA R: CGCCTGCCCCTGAACTATC	121	96.4	Denman & McSweeney (2006)
*Butyrivibrio fibrisolvens*	F: TAACATGAGAGTTTGATCCTGGCTC R: CGTTACTCACCCGTCCGC	136	93.9	Denman & McSweeney (2006)

### Statistical analysis

All the statistical analyses were conducted using the PROC MIXED procedure of SAS 9.2 (2002; SAS Institute, Cary, NC, USA). The data on populations of total bacteria and ruminal cellulolytic bacteria were converted to a logarithm base before statistical analysis. Data from urea hydrolysis, Shannon–Weiner index and bacterial populations were analyzed with urea, AHA, incubation time, and urea × AHA interaction included in the model as fixed effects. Data from separate days were used as repeated measures. Orthogonal polynomial contrasts were performed to compare the treatments. All data were presented as least squares means, and significant differences were declared at *P* < 0.05.

## Results

### Inhibition of urea hydrolysis and ammonia formation

The urea-N concentration could not be detected in the treatment with urea supplementation only by 1 h postincubation, but it gradually decreased until 6 h of incubation in the treatment with both urea and AHA supplementation (Table 2). The concentration of NH_3_-N was increased by urea supplementation through the whole incubation (*P* < 0.05), but it was numerically decreased by AHA addition within 2 h of incubation (significantly in 1 and 2 h). Urease activity was stimulated by urea supplementation within 6 h of incubation (*P* < 0.01, apart from 1 h), but it was numerically inhibited by AHA addition within 2 h of incubation (significantly in 2 h). Additionally, urease activity of U2UI450 treatment was inhibited by 51.7% at 1 h after incubation, and by 35.7% at 2 h after incubation, compared to that in U2UI0 treatment. After incubation of 12 h, there was no statistical differences on MCP concentration among treatments.

**Table 2:** Effects of urea and urease inhibitor supplementation on temporal changes of ammonia-nitrogen (NH_3_-N) and urea-nitrogen (Urea-N) concentrations, urease activity, and microbial crude protein (MCP) after 12 h of incubation in vitro.

Item	Culture time (h)	Treatment^x^				SEM^y^	*P*-Value		
		U0UI0	U0UI450	U2UI0	U2UI450		Urea	AHA	Urea × AHA
NH_3_-N concentrations (mg/100 ml)	0.5	18.64^b^	17.94^b^	21.75^a^	19.99^a^	0.971	0.03	0.24	0.60
	1	16.00^c^	15.62^c^	26.72^a^	18.34^b^	1.338	<0.01	0.01	0.02
	2	17.50^c^	17.24^c^	28.97^a^	24.33^b^	0.627	<0.01	<0.01	<0.01
	4	17.91^b^	16.09^b^	24.30^a^	24.49^a^	2.060	<0.01	0.71	0.64
	6	12.74^b^	15.54^b^	21.95^a^	23.85^a^	2.012	<0.01	0.28	0.83
	12	17.70^b^	16.32^b^	28.09^a^	26.57^a^	1.315	<0.01	0.30	0.96
Urease activity (nmol/min/mg)	0.5	2.26^c^	1.91^c^	4.92^a^	3.10^b^	0.511	0.01	0.08	0.19
	1	2.28^b^	1.58^b^	4.97^a^	2.40^b^	0.908	0.06	0.07	0.27
	2	2.56^c^	1.80^d^	5.16^a^	3.32^b^	0.540	<0.01	0.04	0.32
	4	2.66^b^	1.67^c^	4.45^a^	4.04^a^	0.388	<0.01	0.21	0.34
	6	1.50^b^	1.58^b^	3.42^a^	3.26^a^	0.152	<0.01	0.80	0.47
	12	1.58^b^	2.40^b^	3.34^a^	2.66^a^	0.414	0.07	0.87	0.14
Urea-N concentrations (μmol)	0.5	0	0	44.20	45.10	0.199	–	0.56	–
	1	0	0	0	44.63	0.702	–	<0.01	–
	2	0	0	0	37.74	0.834	–	<0.01	–
	4	0	0	0	31.45	1.921	–	<0.01	–
	6	0	0	0	17.57	1.814	–	<0.01	–
	12	0	0	0	0	0	–	–	–
MCP (mg/100 ml)	12	1.13	1.13	1.21	1.19	0.074	0.40	0.91	0.90

**Notes:**

x Treatments consisted of substrate (U0UI0), substrate with AHA addition (U0UI450), substrate with urea supplementation (U2UI0), and substrate with both AHA and urea supplementation (U2UI450).

y SEM is standard error of the means.

a–c Means treatments are different significantly in the same row.

### Alteration of ruminal bacterial diversity level

**Figure 1:**
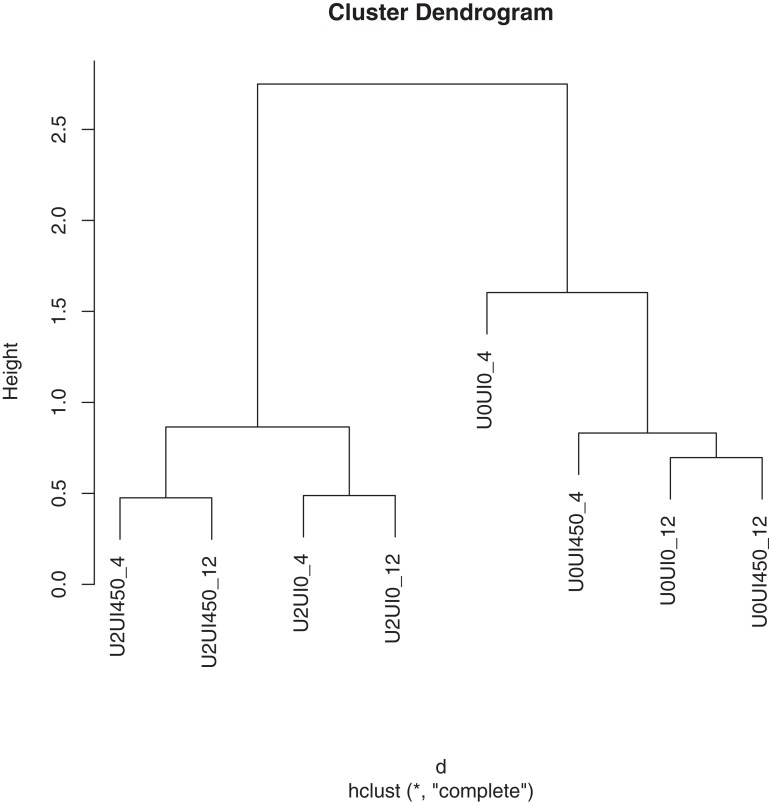
Effects of urea and urease inhibitor (acetohydroxamic acid, AHA) supplementation on bacterial diversity at 0, 4, and 12 h of incubation analysis by DGGE. Treatments without urea supplementation were grouped together to form a cluster (U0), which was separated from the treatments with urea supplementation (U2). Treatments with urea supplementation were further separated according to the AHA addition (U2UI0 *vs.* U2UI450). The ruminal bacterial profiles were separated by ammonia release rate when urea was supplemented, according to the DGGE and hierarchical cluster analysis.

To verify the observed diversity variation among treatments at different incubation stages, the hierarchical cluster analysis was performed using the pooled samples (Fig. 1). Treatments without urea supplementation were grouped together to form a cluster (U0), which was separated from the treatments with urea supplementation (U2). Treatments with urea supplementation were further separated according to the AHA addition (U2UI0 *vs.* U2UI450). The ruminal bacterial profiles were separated by ammonia release rate when urea was supplemented, according to the DGGE and hierarchical cluster analysis. The Shannon–Weiner index showed that treatments with urea supplementation had lower (*P* < 0.05) diversity of predominant species, while AHA addition (U2UI450) could increase (*P* < 0.05) the bacterial diversity.

### Changes of ruminal cellulolytic bacterial abundances

Interactive effects between urea and AHA supplementation were noticed on total bacterial population within 4 h of incubation (*P* < 0.01). After incubation of 12 h, total bacteria population of U2UI0 treatment was lower than other treatments (*P* < 0.05) (Table 3). Interactive effects of urea and AHA supplementation were found on populations of *R. flavefaciens* (*P* = 0.03) and *Butyrivibrio fibrosolvens* (*P* < 0.01) at 0.5 h of incubation, and populations of these two bacteria in treatment of U2UI0 were lower than their populations in treatment of U2UI450 at 0.5, 4, and 12 h of incubation. For *F. succinogenes*, urea supplementation decreased its population at 0.5 h of incubation (*P* = 0.05), but AHA addition increased (*P* = 0.03) it. The *F. succinogenes* population of U2UI0 treatment was lower than other treatments (*P* < 0.05) at 0.5 h of incubation.

**Table 3:** Effects of urea and urease inhibitor supplementation on the populations of ruminal cellulolytic bacteria at 0.5, 4, and 12 h of incubation in vitro.

Item	Culture time (h)	Treatment^x^				SEM^y^	*P*-Value		
		U0UI0	U0UI450	U2UI0	U2UI450		Urea	AHA	Urea × AHA
Total bacteria (log_10_ copies per ml)	0.5	8.22^a^	7.96^b^	7.77^b^	7.97^b^	0.085	0.01	0.74	0.01
	4	8.11^b^	8.35^a^	8.29^b^	8.14^b^	0.067	0.87	0.53	0.01
	12	8.04^a^	8.19^a^	7.93^b^	8.08^a^	0.079	0.16	0.06	0.95
*Ruminococcus albus* (log_10_ copies per ml)	0.5	5.84^a^	5.95^a^	5.50^b^	5.65^b^	0.080	0.02	0.09	0.75
	4	5.41	5.48	5.42	5.29	0.083	0.24	0.67	0.19
	12	5.11	5.12	4.85	4.94	0.131	0.09	0.69	0.78
*Ruminococcus flavefaciens* (log_10_ copies per ml)	0.5	5.12^a^	4.94^a^	4.32^b^	4.62^b^	0.108	<0.01	0.56	0.03
	4	4.31^b^	4.64^a^	4.48^b^	4.54^b^	0.096	0.68	0.03	0.13
	12	3.97^a^	3.87^a^	3.29^b^	3.54^b^	0.130	<0.01	0.51	0.15
*Fibrobacter succinogenes* (log_10_ copies per ml)	0.5	7.03^a^	7.06^a^	6.75^b^	7.05^a^	0.081	0.05	0.03	0.07
	4	7.13	7.13	7.04	7.03	0.050	0.06	0.90	0.92
	12	7.14	7.10	6.89	6.87	0.102	0.04	0.71	0.98
*Butyrivibrio fibrisolvens* (log_10_ copies per ml)	0.5	7.82^a^	7.68^b^	7.47^b^	7.56^b^	0.037	<0.01	0.49	<0.01
	4	7.55^b^	7.66^a^	7.48^b^	7.56^b^	0.033	0.01	<0.01	0.69
	12	7.67^b^	7.74^a^	7.52^b^	7.66^b^	0.056	0.05	0.07	0.57

**Notes:**

x Treatments consisted of substrate (U0UI0), substrate with AHA addition (U0UI450), substrate with urea supplementation (U2UI0), and substrate with both AHA and urea supplementation (U2UI450).

y SEM is standard error of the mean.

a,b Means treatments are different significantly in the same row.

## Discussion

Rumen simulation systems have been used in the evaluation of rumen fermentation manipulation and feeds nutrients degradation in order to avoid the use of animals or decrease study costs (Hristov et al., 2012). In this study, we invented a dual-flow continuous rumen simulation system (a batch culture system) with real-time monitoring. We have previous demonstrated that the conditions of microbial fermentation in this system were similar to those in the rumen of dairy cows (Jin et al., 2016; Shen et al., 2012), making it a powerful and practical tool for the study of rumen microbes or fermentation. Therefore, we can illuminate the influence of hydrolysis rate of urea on rumen fermentation parameters, ruminal bacterial diversity level, and cellulolytic bacteria abundance in vitro.

Urea accompanied with urease inhibitor is usually used as a dietary supplement to provide degradable nitrogen, and acts as a solution for the rapid hydrolysis of urea in rumen. In this study, urea hydrolysis and ammonia formation were successfully inhibited by the interaction of urease inhibitor (AHA) and urea supplementation, which was in agreement with previous in vivo and in vitro studies (Jones & Milligan, 1975; Makkar et al., 1981). Brent, Adepoju & Portela (1971) reported that the linear or quadratic components of ammonia release are strongly associated with the addition of AHA. The doses of AHA between 5 and 10 μg/ml can reduce the initial velocity of ammonia release by 50% using in vitro fermentation with 233 μg/ml of urea supplementation (Brent, Adepoju & Portela, 1971). In our study, addition of six μg/ml AHA in combination with 266 μg/ml urea reduced urease activity by 51.7%, which is consistent with previous results. The inhibition of urease activity in rumen by AHA is time-limited (within 2 h) and not complete (51.7% maximal inhibitory efficiency), and the remaining urease can still hydrolyze urea to ammonia for microbial protein synthesis (Upadhyay, 2012). In fact, both urease activity and the extent of AHA inhibition decreased over the incubation period, because urease enzymes exhibit simple Michaelis–Menten behavior, while urea concentration is proposed to be important for regulating urease activity (Krajewska, 2009). The inhibition of urease activity by AHA happened within 2 h was consistent with ammonia exploration from urea, and which potentially prevents the ammonia toxic to the cows.

The aim of controlling the release rate of ammonia from dietary urea hydrolysis is to allow more efficient incorporation of nitrogen into ruminal microbial protein, which is a major contributor to the available metabolizable protein for intestinal digestion and absorption in ruminants (Berends et al., 2014). Unfortunately, slowing the rate of hydrolysis of urea did not result in more efficient incorporation of nitrogen into microbial protein in the present study. According to previous reports, the optimum rumen NH_3_-N concentration required to support maximum synthesis of microbial protein was 12.8 mg/100 ml in lactating Holstein cows fed a corn silage-based diet with graded levels of urea (Boucher et al., 2007). The optimum NH_3_-N concentration based on maximum microbial growth is 3.7 mmol (approximately 6.29 mg/100 ml) using in vitro fermentation technique (Satter & Slyter, 1974). In this study, the high CP level of the diet used potentially supplied an adequate amount of ammonia (more than 12.74 mg/100 ml in U0UI0 treatment) for microbial protein synthesis, which may have resulted in the similar MCP content across treatments.

The rumen microbial community inhabiting in rumen is characterized by high population density, broad diversity and complex interactions (Shi et al., 2008). It was reported that ruminal bacterial diversity could be influenced by nitrogen levels, under different ammonia concentrations using in vitro batch culture (Wang et al., 2015), which may be the result of the fact that populations of major ruminal bacteria is dose-dependent on ammonia concentration (Mehrez, Orskov & McDonald, 1977). In current study, a significant clustering effect was observed between with urea and without urea supplementation, which may be the result of that the potential NH_3_-N (a maximum of 11.33 mg in theoretical) released from 0.02 g of dietary urea hydrolysis altered the level of nitrogen in cultural solutions.

Ruminal cellulolytic bacteria are key players in the dynamics of rumen microbial community, and these bacteria utilize NH_3_-N to synthesize microbial protein in the rumen (Pengpeng & Tan, 2013). The growth of those ruminal cellulolytic bacteria depends on the dose-effect of NH_3_-N concentration in the rumen (Amaya et al., 2005; Purich, 1998). Therefore, enough ammonia from urea hydrolysis and dietary protein degradation inhibits their populations. However, AHA addition slows down the release rate of ammonia from urea. AHA addition can limit the asynchronous supplement of nitrogen and energy in some extent and lead to lessen the inhibition of cellulolytic bacterial populations (Yu, Hart & Du, 2008). Considering the growth cycle of cellulolytic bacteria, it is not surprising that some cellulolytic bacterial populations in U2UI450 treatment are higher than that in U2UI0 treatment until the end of incubation process, though the interaction between urea and AHA appears occurs in the first few hours of incubation. Furthermore, U2UI450 significantly increased the level of bacterial diversity, and which is consistent to the publication (Jin et al., 2016). In general, microbial ecosystems with greater microbial diversity are more stable (Lacombe et al., 2009), and a stable rumen ecosystem is beneficial to the host. Urease inhibitor supplementation would be benefit to the ruminal bacterial community while urea were supplemented. However, it was reported that the rumen microbes could possibly adapt to the addition of AHA in the diet with time, which could result in ammonia concentration rebounding again (Streeter et al., 1969). Therefore, long-term animal trial will be helpful to evaluate the AHA effects on rumen microbes, and improved urease inhibitors based on AHA need to be synthesized in our following works.

## Conclusion

In conclusion, urease inhibitor (AHA) addition significantly decreased the rate of ammonia formation when urea was supplemented. Slow release of ammonia induced by AHA influenced the ruminal bacterial diversity level (depend on the result from DGGE cluster) and lessened the inhibition of total bacteria growth at the incubation of 12 h and *F. succinogenes* at the incubation of 0.5 h.

## Supplemental Information

10.7717/peerj.5475/supp-1 Supplemental Information 1 DGGE gel.

10.7717/peerj.5475/supp-2 Supplemental Information 2 Raw data include DGGE fingerprint, the raw data of qPCR, MCP, NH_3_N, Urea, and Urease.

## Additional Information and Declarations

### Data Availability

The following information was supplied regarding data availability: The raw data are provided in the Supplemental Files.
