# Supplementary figures and images for: Influence of hydrolysis rate of urea on ruminal bacterial diversity level and cellulolytic bacteria abundance in vitro
Source: PeerJ. 2018 Aug 17;6:e5475. doi: 10.7717/peerj.5475 (PMC6100864; doi:10.7717/peerj.5475)

$U0UI0$     $U0UI450$  ↓  $U2UI0$     $U2UI450$  ↓  $U0UI0$     $U0UI450$  ↓  $U2UI0$     $U2UI450$  ↓  $U0UI0$     $U0UI450$  ↓  $U2UI0$     $U2UI450$  ↓

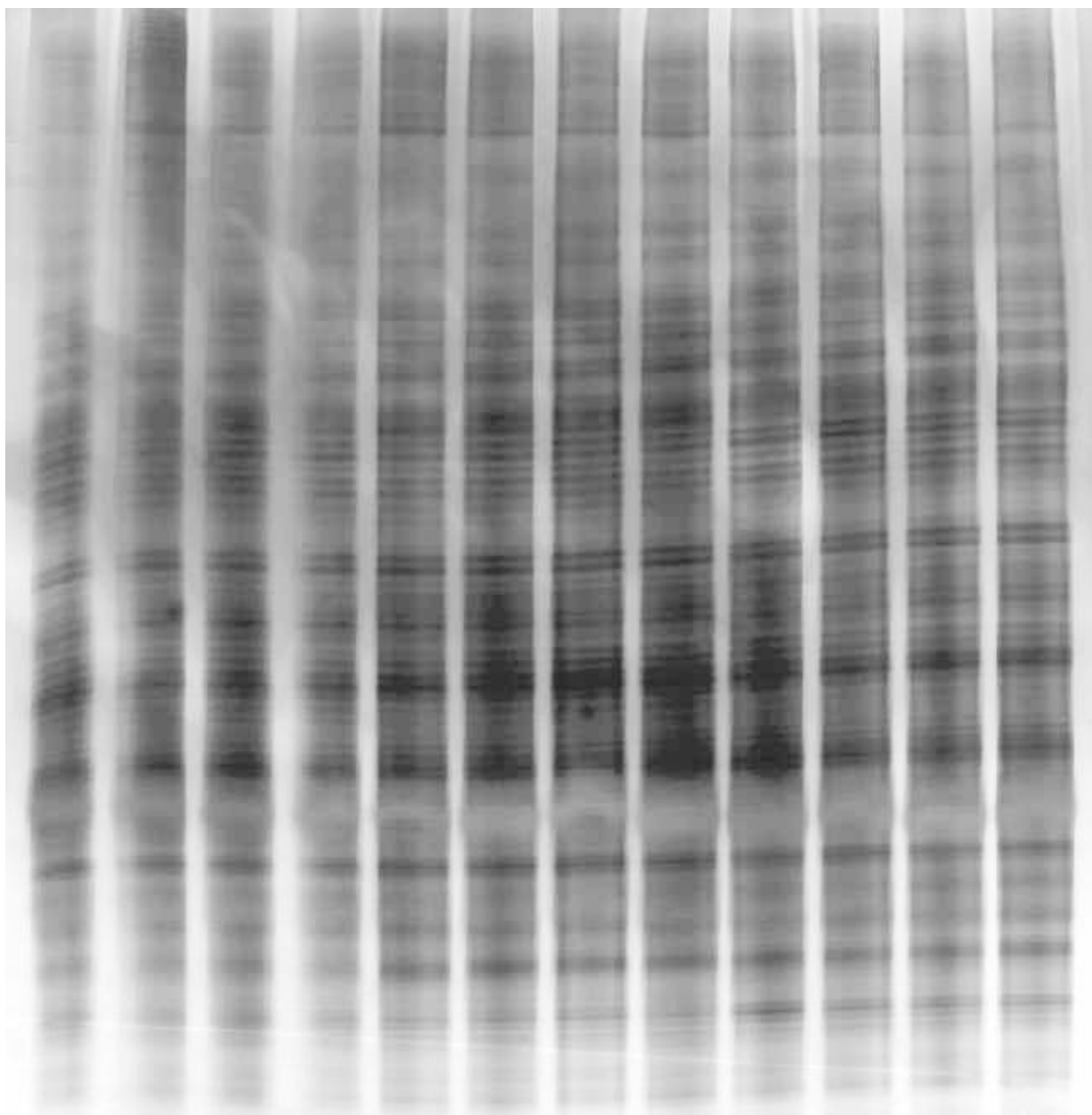

← 0.5 h → ← 4 h → ← 12 h →

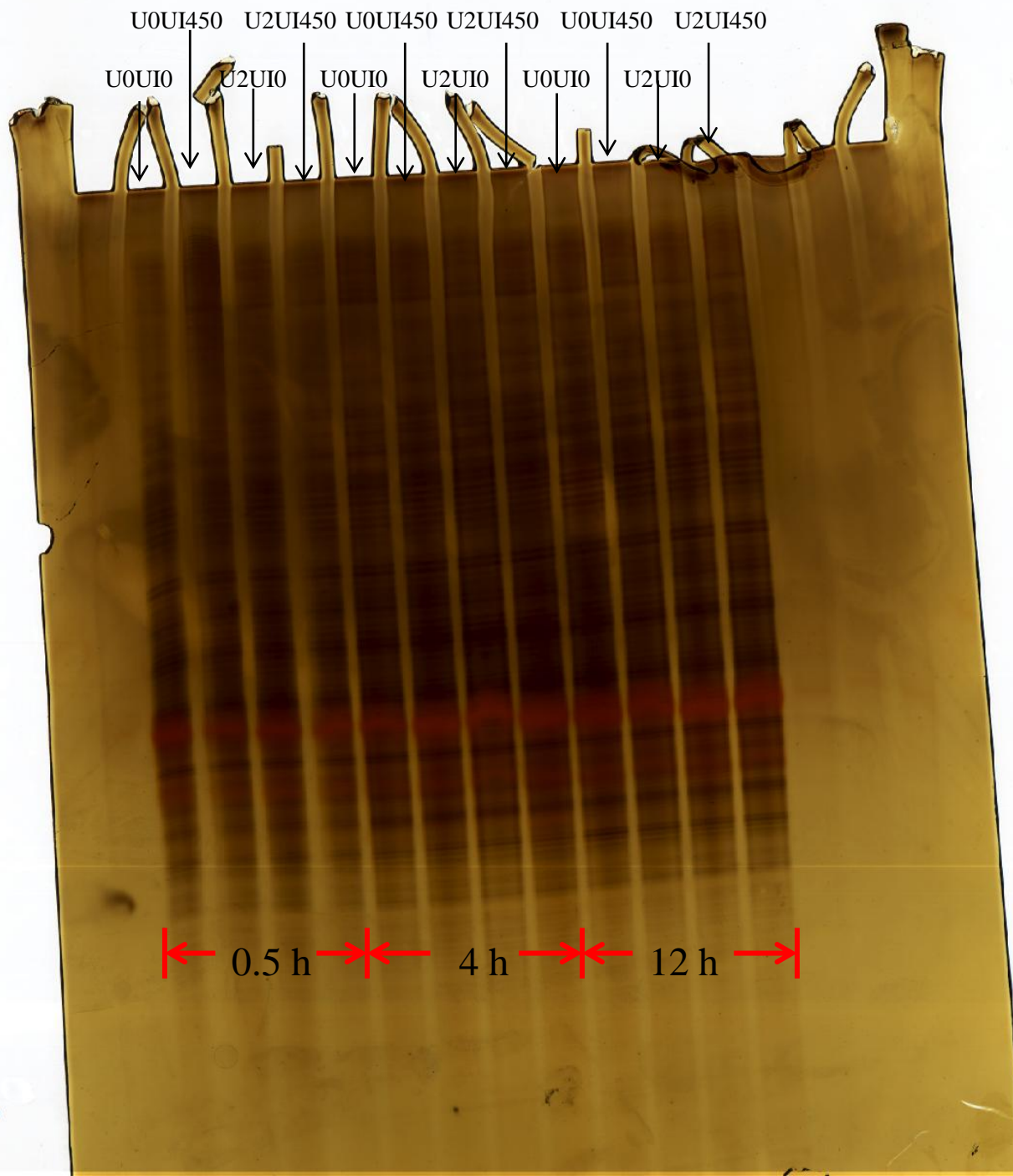

Supplement: Supplemental Information 1 [file peerj-06-5475-s001.pdf]
